# A Thermostable Bilirubin-Oxidizing Enzyme from Activated Sludge Isolated by a Metagenomic Approach

**DOI:** 10.1264/jsme2.ME16106

**Published:** 2016-11-23

**Authors:** Nobutada Kimura, Yoichi Kamagata

**Affiliations:** 1 Bioproduction Research Institute, National Institute of Advanced Industrial Science and Technology (AIST)Tsukuba, Ibaraki 305–8566Japan

**Keywords:** bilirubin oxidase, metagenomics, multicopper oxidase, thermostable enzyme

## Abstract

A gene coding for a multicopper oxidase (BopA) was identified through the screening of a metagenomic library constructed from wastewater treatment activated sludge. The recombinant BopA protein produced in *Escherichia coli* exhibited oxidation activity toward 2,2′-azino-bis-(3-ethylbenzothiazoline-6-sulfonate) (ABTS) in the presence of copper, suggesting that BopA is laccase. A bioinformatic analysis of the *bopA* gene sequence indicated that it has a phylogenetically bacterial origin, possibly derived from a bacterium within the phylum *Deinococcus-Thermus*. Purified BopA exhibited maximum activity at pH 7.5 with bilirubin as its substrate and was found to be active over a markedly broad pH range from 6 to 11. It also showed notable thermostability; its activity remained intact even after a heat treatment at 90°C for 60 min. This enzyme is a thermostable-bilirubin oxidase that exhibits markedly higher thermostability than that previously reported for laccases.

Multicopper oxidases (MOCs) catalyze the introduction of one or two oxygen atoms into substrate molecules. MOCs have been utilized as important biocatalysts for craft pulp biobleaching, the synthesis of secondary metabolites, and biodegradation of toxic compounds (51) as well as in other applications. The discovery of novel proteins with MOC-like activity from biological resources has been attracting increasing attention (6, 9, 15, 32, 39, 46, 49). MOCs are widely distributed among eukaryotes and prokaryotes, and functional and structural studies have been performed on these enzymes (42). Current commercial MOCs are derived from fungus-derived enzymes (16, 44, 47). However, some fungal enzymes are deactivated at elevated temperatures (37).

In an attempt to discover MOCs with novel catalytic potential, screening has been conducted to identify microorganisms with the ability to catalyze the oxidation of substrates by mono- or dioxygenation. However, since more than 99% of microbes in the environment are not easily cultivated (34), metagenomic approaches may circumvent culture-based investigations and comprehensively capture a diverse array of target gene families (14). Novel biocatalysis-relevant enzymes originating from environmental microbial communities have been discovered using a metagenomic approach (22, 51). For example, a gene encoding a novel MOC with laccase activity was identified through the activity-based functional screening of a metagenomic library from mangrove soil (51), and a novel MOC was also identified through the activity screening of a metagenome expression library from bovine rumen microflora (3). Thus, metagenomic approaches are regarded as a tool for examining enzymes from environmental samples.

In coking plants, coals are converted into coke and organically polluted water is formed during this process. Coke plant wastewater, which is produced by the quenching of hot coke and washing the gas produced in coking plants, contains various organic pollutants such as phenols, mono- and polycyclic nitrogen-containing aromatics, oxygen- and sulfur-containing heterocyclic compounds, and polycyclic aromatic hydrocarbons (43). It has been suggested that as yet unidentified, but functionally interesting enzymes exist in activated sludge left over from coke plant wastewater treatments. We recently reported that a metagenomic library of coke plant wastewater harbored a number of unknown organisms and also isolated and characterized novel types of aromatic compound-oxidizing enzymes (21).

In the present study, we isolated and characterized a new bilirubin oxidase (BOD) with laccase activity from a metagenomic library, designated hereinafter as BopA. The metagenomics of activated sludge from a coke plant were used to isolate a gene encoding a biocatalytic enzyme (21). BOD catalyzes the oxidation of bilirubin to biliverdin (5) and is used clinically to measure the levels of total and conjugated bilirubin in serum (7, 23, 33). BopA is a thermostable BOD that exhibits markedly higher thermostability than that reported for laccases; BopA appears to be the most thermostable BOD identified to date.

## Materials and Methods

### Strains and growth conditions

*E. coli* cultures were grown at 37°C on Luria-Bertani (LB) agar or in LB broth supplemented with the appropriate antibiotics. The following antibiotic concentrations were used for the *E. coli* strains: chloramphenicol, 12.5 μg mL^−1^; ampicillin, 100 μg mL^−1^. *E. coli* strain EPI300 was obtained from Epicentre (Madison, WI, USA). *E. coli* strain BL21 (DE3) was obtained from Takara Bio (Otsu, Japan).

### Chemicals

Bilirubin was purchased from Wako (Osaka, Japan). Molecular mass markers for SDS-PAGE were obtained from Bio-Rad (Tokyo, Japan). Restriction enzymes and DNA ligase were purchased from Toyobo (Osaka, Japan).

### DNA manipulations, sequencing, and computer analysis

Fosmid DNA purification was performed with the QIAprep Spin Miniprep Kit (QIAGEN, Valencia, CA, USA) in accordance with the manufacturer’s instructions. Restriction endonuclease digestion, DNA ligation, plasmid DNA transformation, agarose gel electrophoresis, and other standard recombinant DNA techniques were performed using standard methods described by Sambrook *et al.* (38). DNA sequencing was conducted with an Applied Biosystems 373A automated DNA sequencer (Applied Biosystems, Tokyo, Japan). DNA sequences were analyzed with BLAST and ORF finder programs provided by the National Center for Biotechnology Information (www.ncbi.nlm.nih.gov) (1).

### DNA preparation and library construction

Activated sludge was collected from the aeration tank of a wastewater treatment facility in Japan. The sample was immediately stored at −80°C and simultaneously subjected to metagenomic DNA isolation. DNA was extracted from the sludge as previously described using a sodium dodecyl sulfate and proteinase K treatment (20). DNA was further purified for cloning into a fosmid following the methods of Rondon *et al.* (35). The size of extracted DNA was examined by agarose gel electrophoresis. Metagenomic libraries using DNA extracted from the activated sludge was constructed using the commercial fosmid vector, Copy Control™ pCC1Fos (Epicentre). The library was constructed by DNA size fractionation, a clean-up of metagenomic DNAs, and subsequent ligation into a fosmid vector. The ligation mixture was then packaged into lambda phages using MaxPlax Lambda Packaging Extracts (Epicentre). The packaged library was transfected into *E. coli* EPI300. *E. coli* transformants were selected on LB agar supplemented with chloramphenicol. The presence of recombinant plasmids and polymorphisms in insert DNA were examined by agarose gel electrophoresis of the *Eco*RI digestion of purified plasmids from randomly selected *E. coli* transformants.

### Screening of an active clone

In order to select a clone exhibiting laccase activity from the stored library, metagenomic clones in *E. coli* were cultured on LB agar supplemented with chloramphenicol at 37°C overnight. The number of clones per plate was adjusted to approximately 500 by dilution of the library stock, and plates were incubated at 37°C overnight. Soft agar solution containing 0.7% agar and 0.05 mM 2,4-dinitrophenol was overlaid on the plate, which was then incubated at 37°C for 2 d. During the library storage process, we selected a laccase active clone that degrades 2,4-dinitrophenol.

### Phylogenetic analysis

The BLAST program was used to compare BopA protein sequences with bacterial protein sequences in the NCBI database (http://www.ncbi.nlm.nih.gov/BLAST) (1). Amino acid sequences were aligned using ClustalW (48), and any gap-containing columns in the alignment were removed. Phylogenetic trees were constructed by the neighbor-joining method, and bootstrap analyses for 1,000 replicates were performed (36).

### Cloning of the *bopA* gene

The *bopA* gene was amplified by PCR with the primers 24DNPoxi F-3 (5′-GGAATTCCATATGACAAAACTAACGCGACGC-3′) and 24DNPoxi R-3 (5′-CGGGATCCTCAGGCCCGAATCTCGAAAT TA-3′) with the selected fosmid (E371) isolated from an active transformant as a template, and with Ex *Taq* DNA polymerase (Takara). Amplification conditions was as follows: 94°C for 20 s; 30 cycles of 94°C for 20 s, 55°C for 30 s, 72°C for 1 min; 72°C for 7 min. PCR products were purified using the QIAquick PCR purification kit (QIAGEN, Hilden, Germany). PCR products were then ligated with the high expression vector pET19b (Novagen, Madison, WI, USA) in order to construct the plasmid pBop101, which was introduced into *E. coli* BL21 (DE3) (Invitrogen, Carlsbad, CA, USA).

### Expression and purification of the recombinant BopA protein

*E. coli* BL21 (DE3) cells harboring the plasmid pETBopA were incubated at 37°C overnight on LB broth supplemented with 12.5 μg mL^−1^ ampicillin and 0.25 mM CuSO_4_. When cultures reached an A600 of 0.6, 1 mM isopropyl-β-D-galactopyranoside was added, and the incubation continued for 24 h. Cells were collected by centrifugation (4,000×*g*, 4°C, 15 min), resuspended in 5 mL of 50 mM NaH_2_PO_4_ solution, and 50 μL of 100 mM CuSO_4_ was added. After centrifugation at 7,000 rpm for 3 min, cell suspensions were suspended in 50 mM NaH_2_PO_4_ solution and sonicated four times for 1 min. Cell lysates were centrifuged at 6,000×*g* at 4°C for 10 min, and His_6_-tagged enzymes were purified at 25°C on a Ni-NTA spin column (QIAGEN). After the columns had been washed with 600 μL Wash Buffer (QIAGEN), recombinant enzymes were eluted with 200 μL Elution Buffer (QIAGEN). SDS-PAGE was performed on 12% (v/v) acrylamide gels, as described by Laemmli (24), in a Bio- Rad Mini Protean system. Protein concentrations were measured according to Bradford with bovine serum albumin as the standard (4).

### Measurement of enzymatic activity

We assayed the enzymatic activity of the isolated BopA using bilirubin as its substrate. The monitoring of enzymatic activity was performed by spectrophotometric measurements of the oxidation of bilirubin at 450 nm. Unless otherwise specified, the reaction mixture contained 50 mM Britton and Robinson buffer (pH 7.5), 5 μg of the substrate, and 1 to 3 mU of the enzyme in a total volume of 100 μL. The copper ion dependency of enzymatic activity was assessed at 25°C in a reaction mixture containing 100 mM sodium acetate buffer (pH 4.5), 50 mM bilirubin as the substrate, 15 μM-1.0 mM CuSO_4_, and 1–3 mU of the enzyme in a total volume of 200 μL. The pH dependency of enzymatic activity was assessed at 25°C in a reaction mixture containing 50 mM Britton and Robinson buffer (50 mM boric acid, 50 mM acetic acid, and 50 mM phosphoric acid), 50 mM bilirubin as the substrate, 0.5 mM CuSO_4_, and 1–3 mU of the enzyme in a total volume of 200 μL at pH from 2.5 to 9.5. The temperature dependency of enzymatic activity was evaluated in a reaction mixture containing 50 mM Britton and Robinson buffer (pH 7.5), 50 mM bilirubin as the substrate, 0.5 mM CuSO_4_, and 1–3 mU of the enzyme in a total volume of 200 μL at temperatures from 0°C to 90°C. The thermostability of the enzyme was measured in a reaction mixture containing 50 mM Britton and Robinson buffer (pH 7.5), 50 mM bilirubin as the substrate, 0.5 mM CuSO_4_, and 1–3 mU of the enzyme in a total volume of 200 μL at 0, 10, 20, 30, 40, 50, 60, 70, 80, and 90°C by incubating the enzyme in microtubes for 30 min, followed by chilling the tubes in an ice-water bath. In order to ensure temperature control, experiments were performed using a thermal cycle. The oxidation of bilirubin was monitored spectrophotometrically at 450 nm (e450=41 mM^−1^cm^−1^ for bilirubin). The reaction was started by the addition of the enzyme.

### Molecular modeling

A search in the Protein Data Bank for proteins of known structures homologous to BopA yielded two entries: 1w8eA from *Bacillus subtilis* and 2yxwB from *E. coli*, which exhibited 26.5% and 31.2% sequence identities with BopA, respectively. We used 1w8eA as a suitable template for modeling. The structural alignment of the BopA and 1w8eA sequences was achieved with GenTHREADER (18, 27) and used to retrieve a BopA model from the Swiss-Model server (2, 13, 40). A Ramachandran plot of the predicted BopA structure yielded all residues in the favored regions indicative of a model of good quality.

#### Nucleotide sequence accession numbers

The nucleotide sequences reported in this study were deposited in the GenBank database with the accession number AB830740.

## Results

### Gene cloning and genetic characterization of the *bopA* gene

A clone designated E371 containing the gene of an enzyme exhibiting laccase activity, a bilirubin-oxidizing protein, the BopA protein (GenBank/EMBL/DDBJ accession number AB830740), was identified from approximately 100,000 metagenomic fosmid clones by screening a fosmid metagenomic library of wastewater treatment activated sludge on indicator plates supplemented with 2,4-dinitrophenol (for details, see “Materials and Methods”). The sequence analysis of the 19,576-bp cloned fragment predicted 15 open reading frames (ORFs) using the ORF finder for gene prediction (Table 1). A sequence analysis through BlastP of the deduced polypeptide sequences of the *bopA* gene product revealed similarities to laccases or polyphenol oxidases. The deduced product of the BopA protein (528 amino acids) belonged to the family of MOCs, and exhibited similarities to the spore copper-dependent laccase from *B. subtilis* (27% identity). The BopA protein also showed homology (28% identity) to the BOD of *Myrothecium verrucaria*. Molecular weight was calculated as 58,486 Da, and theoretical pI as 5.33 using the ProtParam tool maintained by the Swiss Institute of Bioinformatics (http://www.expasy.org/tools/protparam.html) (12).

**Fig. 2:**
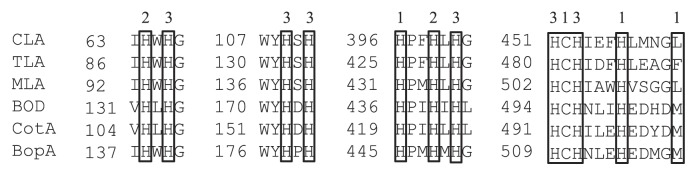
Comparison of amino acid sequences of potential copper coordination sites in multicopper oxidases. BopA in metagenomic clone E371, bilirubin oxidase of sludge metagenomic clone E371 (NCBI entry AB830740); CotA, *B. subtilis* CotA (NCBI entry NP_388511); BOD, *M. verrucaria* BOD (NCBI entry Q12737); CLA, *Coprinus cinereus* laccase (NCBI entry 1HFUA); TLA, *Trametes villosa* laccase (NCBI entry AAB47735); MLA, *Melanocarpus albomyces* laccase (NCBI entry CAE00180). The numbers 1, 2, and 3 indicate the potential coordination sites for type 1, 2, and 3 copper ions, respectively. The amino acid residues presumed to be involved in binding copper are boxed.

**Fig. 3:**
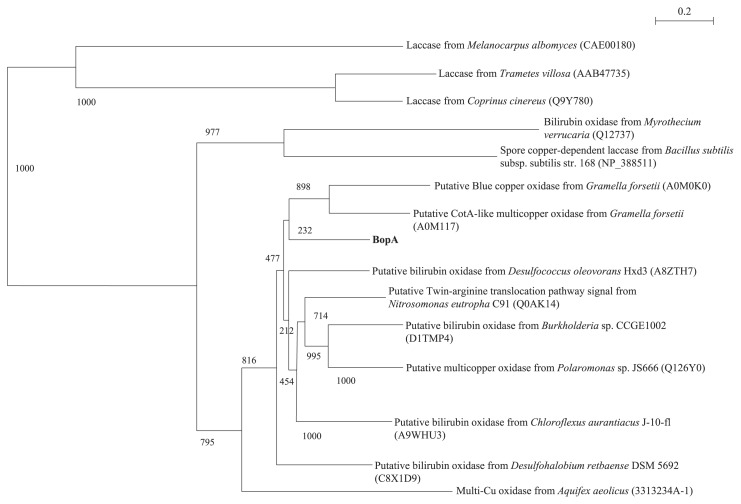
Phylogenetic tree of the BopA protein aligned with sequences of the most closely related proteins from a BLAST search. The neighbor-joining method was used to construct phylogenetic trees. The bootstrap value attached to each branch is a measure of confidence in this branch. Protein and strain abbreviations and accession numbers: BopA in metagenomic clone E371, bilirubin oxidase of sludge metagenomic clone E371 (AB830740).

Sequence alignment of the BopA protein with the reported multicopper family of enzymes clearly indicated that the BopA protein is a member of the multicopper family of enzymes (Fig. 2). Comparisons of the putative amino acid sequence of the BopA protein with those of the other blue copper proteins indicated that the BopA protein has four consensus domains that are assumed to be copper ligands. As shown in Fig. 2, the amino acid sequence of BOD contains consensus domains for the copper ligands of all types (I, II, and III), which were revealed by crystallography on ascorbate oxidase from zucchini (28). All the homologs identified were aligned using ClustalW, resulting in a phylogenetic tree of MOCs (Fig. 3). In this tree, the BopA protein was positioned close to BOD.

The G+C ratio of the *bopA* gene sequence was calculated as 56%. Highly conserved genes such as those encoding 16S rRNA, DNA polymerase, and RecA may be used to derive phylogenetic inferences of the metagenomic libraries. The genomic fragment carrying the BopA protein contained a putative chromosome partitioning protein, ParB similar to the *Thermus* species, bacteria belonging to the *Deinococcus-Thermus* phylum (23% homology).

### Enzymatic properties and activities

**Fig. 4:**
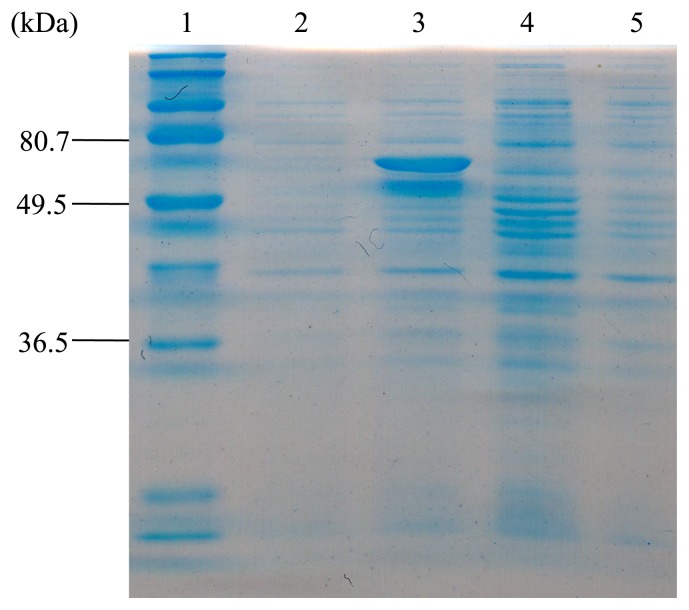
Sodium dodecyl sulfate-polyacrylamide gel electrophoresis of BopA produced in *E. coli* carrying plasmids listed below. Lane 1, standard molecular mass marker; lane 2, pETBopA (non-IPTG-induced); lane 3, pETBopA (IPTG-induced); lane 4, pET19b (non-IPTG-induced); lane 5, pET19b (IPTG-induced).

**Fig. 5:**
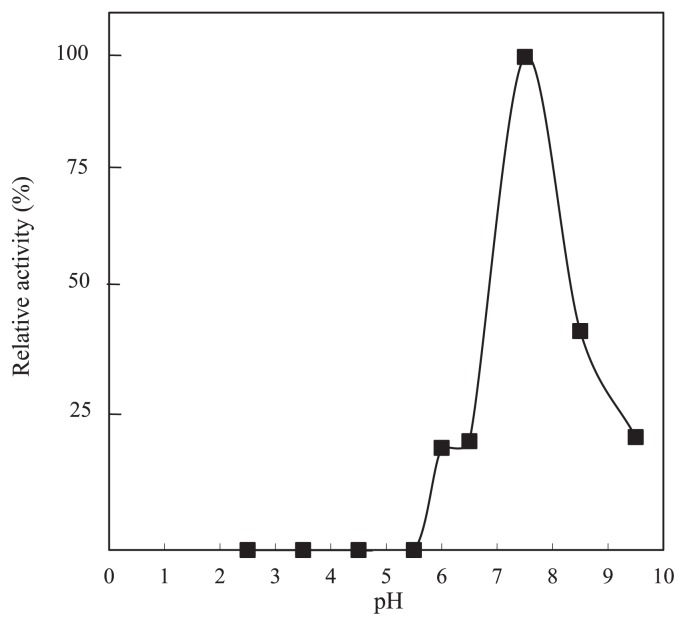
Effects of pH on BopA-catalyzed oxidation of bilirubin.

In order to confirm the supposition that the *bopA* gene encodes BOD, we produced it as a fusion with a hexahistidine (His_6_) tag at the N terminus and investigated its biochemical properties. The *bopA* gene was cloned and overexpressed in *E. coli* BL21 (DE3). Approximately 0.12 mg of the purified His-tagged BopA protein was prepared from cells obtained from a 50-mL culture. A SDS-PAGE analysis revealed that the molecular mass of the purified BopA protein was estimated to be 59 kDa (Fig. 4). The Michaelis constant was assessed from double-reciprocal plots of the initial oxidation rates and concentrations of bilirubin at 37°C and pH 7.0. BopA showed typical Michaelis-Menten kinetics for bilirubin. With bilirubin as the substrate, apparent K_m_ and V_max_ values were 0.0175 mM and 125 μmol min^−1^ mg^−1^, respectively. This enzymatic activity was copper-dependent, and the presence of 0.5 mM CuSO_4_ was optimum for enzymatic activity. The optimum pH for bilirubin oxidation was found to be approximately pH 7.5 (Fig. 5). The recombinant BopA protein produced in *E. coli* exhibited activity to oxidize the classical laccase substrate, 2,2′-azino-bis-(3-ethylbenzothiazoline-6-sulfonate) (ABTS) in the presence of copper.

### Thermostability of recombinant BopA

**Fig. 6:**
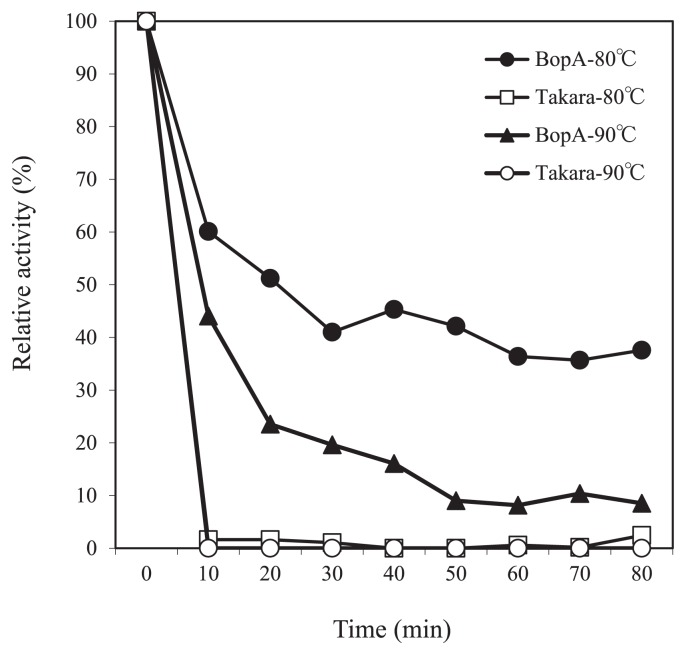
Thermostability assay for the recombinant BopA protein. Closed circles, BopA protein pretreated at 80°C; closed triangles, BopA protein pretreated at 90°C; open squares, *T. tsunodae* K-2593 BOD (Takara) pretreated at 80°C; open circles, *T. tsunodae* K-2593 BOD pretreated at 90°C.

The thermostability of BopA from the metagenomic clone E371 was investigated. In order to assess its thermostability, the enzyme (0.03 mg mL^−1^) was incubated in a mixture of 50 mM Britton and Robinson buffer (pH 7.5) at various temperatures, and residue activity was measured using the standard assay described above. We compared the thermostability of BopA with that of *T. tsunodae* K-2593 BOD (Takara), and found that BopA had lost none of its BOD activity after being heated at 90°C for 60 min, whereas the activity of the *T. tsunodae* enzyme was completely lost (Fig. 6). The aliphatic index of proteins of thermophilic bacteria is known to be significantly higher than that of ordinary proteins (17). Aliphatic index values were calculated using ProtParam by the Swiss Institute of Bioinformatics. The *B. subtilis* endospore component CotA has been identified as a bacterial laccase, and thermostability has so far been reported for the CotA of *B. subtilis* (37). The index for the BopA protein was calculated to be 79.60, while that for CotA was 77.89. The BopA protein has a higher value than that of CotA.

## Discussion

In the present study, we attempted to isolate genes for the laccase enzyme from an activated sludge metagenomic library. Metagenomic technology allows access to uncultivated microbes and provides information regarding microbial communities in the environment. Researchers have been able to identify novel genes or operons from enrichment cultures more frequently in some metagenomic studies than in others (10, 11, 21, 26, 30, 31, 45). In the present study, a gene encoding BOD was identified in a sludge metagenomic library. The results obtained showed that metagenomic technology allows us access to the genes for the laccase enzyme from environmental samples.

**Fig. 7:**
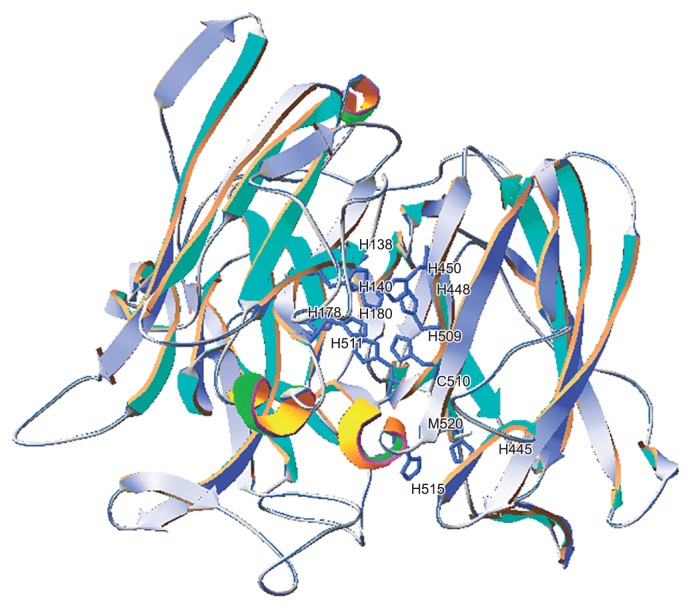
Model of the BopA protein. The overall three-dimensional structure obtained by homology modeling and His residues predicted as copper ligands.

BopA is considered to be a member of the MOC family. BOD contains three types of copper ions: a type I (blue) copper ion, paramagnetic (electron paramagnetic resonance) type II copper ion, and pair of diamagnetic type III copper ions (16, 41). Laccase, ceruloplasmin, and ascorbate oxidase as well as BOD are known to belong to the MOC family and have consensus sequences containing histidine, which suggests the presence of copper ligands in amino acid sequences (28, 29). In order to identify potential residues involved in copper coordination, we generated a three-dimensional model of the BopA protein based on the alignment of its sequence with that of the protein 1w8eA from *B. subtilis*, which exhibited 26.5% sequence identity with the BopA protein (Fig. 7). This model suggested additional copper ligand centers: a type I (blue) copper ion formed by His445, Cys510, H515, and M520, a paramagnetic (electron paramagnetic resonance) type II copper ion formed by His138 and His448, and a pair of diamagnetic type III copper ions formed by His140, His178, His180, His450, His509, and His511.

BopA is a thermostable BOD that exhibits markedly higher thermostability than previously reported for laccases (16, 25, 37, 47). The thermostability of CotA has so far been reported for the CotA of *B. subtilis*, which was inactivated at 65°C for 100 min (37). Thermostability has also been reported for *M. verrucaria* BOD, which rapidly inactivates at 70°C (47). The *bopA* gene was not similar to the previously reported genes for bilirubin oxidation (8, 16, 19, 25, 37, 47, 50). BopA was similar to the putative CotA-like MOC from *Gramella forsetii* (GenBank/EMBL/DDBJ accession number A0M117); however, homology (49% identity) was not high. Only a limited number of homologs have been found in the genome sequence database, indicating that the BopA protein is a member of a relatively small family of bacterial oxidases (Fig. 3).

This study showed that metagenomic technology allows access to unknown genes and enzymes including thermostable enzymes from the environment. The genomic fragment carrying the BopA protein contains a putative chromosome partitioning protein, ParB similar to the *Thermus* species, bacteria belonging to the *Deinococcus-Thermus* phylum. *Deinococcus-Thermus* is one of the dominant bacterial phyla of the hyperthermophiles, indicating that the BopA protein is derived from the hyperthermophiles.

## Figures and Tables

**Fig. 1:**
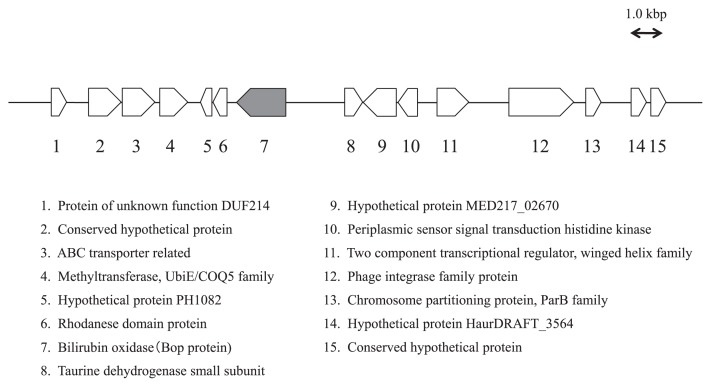
Physical map and gene organization of clone E371. The arrows in the physical map indicate the size, location, and direction of the transcription of ORFs; below are lists of ORFs with the protein to which each is the most similar.

**Table 1:** BLAST results of ORFs identified on metagenomic fosmid clones

No.	ORF		Size (nt^a^)	Possible function	Microorganism	% Identity (no. of identical aa/total no.)
ORF1	2353 ..	3459	1107	Protein of unknown function DUF214	*Nocardioides* sp. JS614	32% (120/372)
ORF2	3499 ..	3789	291	Conserved hypothetical protein	*Listeria monocytogenes* str. 4b H7858	30% (23/76)
ORF3	3518 ..	4354	837	ABC transporter related	*Nocardioides* sp. JS614	48% (135/276)
ORF4	4649 ..	5302	654	Methyltransferase, UbiE/COQ5 family	*Methylococcus capsulatus* str. Bath	54% (100/185)
ORF5	4852 ..	5265	414	Hypothetical protein PH1082	*Pyrococcus horikoshii* OT3	33% (33/100)
ORF6	5320 ..	5775	456	Rhodanese domain protein	*Mycobacterium* sp. JLS	37% (53/143)
*bopA*	5924 ..	7510	1587	Bilirubin oxidase	*Candidatus Desulfococcus oleovorans* Hxd3	52% (288/549)
ORF8	5966 ..	6385	420	Taurine dehydrogenase small subunit	*Roseobacter denitrificans* OCh 114	37% (26/70)
ORF9	9326 ..	9904	579	Hypothetical protein MED217_02670	*Flavobacterium* sp. MED217	65% (116/176)
ORF10	9935 ..	11089	1155	Periplasmic sensor signal transduction histidine kinase	*Roseiflexus castenholzii* DSM 13941	42% (159/374)
ORF11	11086 ..	11772	687	Two component transcriptional regulator, winged helix family	*Roseiflexus castenholzii* DSM 13941	53% (123/228)
ORF12	12388 ..	13395	1008	Phage integrase family protein	*Solibacter usitatus* Ellin6076	41% (127/309)
ORF13	14609 ..	16699	2091	Chromosome partitioning protein, ParB family	*Thermus thermophilus* HB8	23% (60/252)
ORF14	18407 ..	19033	627	Hypothetical protein HaurDRAFT_3564	*Herpetosiphon aurantiacus* ATCC 23779	31% (64/202)
ORF15	19044 ..	19576	534	Conserved hypothetical protein	*Anabaena variabilis* ATCC 29413	26% (47/178)

a nt, nucleotide
