# A Thermostable Bilirubin-Oxidizing Enzyme from Activated Sludge Isolated by a Metagenomic Approach

**DOI:** 10.1264/jsme2.ME16106e

**Published:** 2017-03

**Authors:** Nobutada Kimura, Yoichi Kamagata

**Affiliations:** 1 Bioproduction Research Institute, National Institute of Advanced Industrial Science and Technology (AIST)Tsukuba, Ibaraki 305–8566Japan

Volume 31, no. 4, Page 435–441, 2016

Page 435

Year of “Published online” is incorrect. It should be corrected as follows:

incorrect: 2015

correct: 2016

This correction has been completed on the electronic file of the present paper available at J-STAGE and PMC.

The editorial board expresses sincere apology for the misprinting.

Microbes and Environments editorial board

